# Utilization of marigold leaves (*Tagetes erecta L*.) in rations and their effect on rumen enzyme activity, fermentation parameters, methane emission, and nutrient digestibility *in vitro*

**DOI:** 10.5455/javar.2023.j734

**Published:** 2023-12-31

**Authors:** Chusnul Hanim, Moh. Sofi’ul Anam, Lies Mira Yusiati, Muhsin Al Anas

**Affiliations:** Department of Animal Nutrition and Feed Science, Faculty of Animal Science, Universitas Gadjah Mada, Yogyakarta, Indonesia

**Keywords:** *In vitro*, marigold leaves, nutrient digestibility, rumen fermentation

## Abstract

**Objective:**

This study evaluated the utilization of marigold leaves (MGLs) in rations and their impact on rumen enzyme activity, fermentation parameters, methane (CH_4_) emission, and nutrient digestibility *in vitro*.

**Materials and Methods:**

The experimental diets comprised different proportions of MGL incorporated into the dry matter (DM) rations. Experimental design: The MGL treatments in diets include 0% (MGL-0), 7% (MGL-7), and 14% (MGL-14).

**Results:**

Results indicated that MGL-14 substantially raised (*p <* 0.05) the rumen parameters, including NH_3_–N and microbial protein, total volatile fatty acids, acetate (C_2_), propionate (C_3_), butyrate (C_4_), and the C_2_:C_3_ ratio. In contrast, the MGL-7 and MGL-14 groups experienced a noteworthy reduction (*p <* 0.05) in the total protozoa population. The MGL-7 and MGL-14 treatments also led to a substantial increase in the digestibility of DM, organic matter (OM), and crude fiber (CF) in the rumen. However, they also resulted in a decline (*p <* 0.05) in crude protein (CP) digestibility. The DM and OM total digestibilities were higher (*p <* 0.05) in the MGL-14 and MGL-7 groups. The utilization of MGL did not influence (*p >* 0.05) the ruminal enzyme activities (carboxymethyl cellulase, amylase, protease), cumulative gas production, kinetics, ruminal pH value, CH_4_ and CO_2_ production, total CF, and CP digestibility.

**Conclusion:**

The utilization of MGL until 14% DM in diets can enhance ruminal fermentation parameters and nutrient digestibility *in vitro* without negatively affecting gas production kinetics or ruminal enzyme activities. However, it did not have any impact on CH_4_ production.

## Introduction

Livestock rearing plays a crucial role in meeting the global demand for food. However, in several tropical countries, the production of animals is frequently hindered by the need for more availability and better-quality animal feeds. The scarcity of feedstuffs, particularly protein sources like legumes, cereals, and grains, has led to their high cost and limited accessibility in many regions worldwide. Therefore, it is crucial to explore cost-effective alternative feed sources that are nutritionally rich in essential nutrients and capable of meeting the critical requirements of ruminant animals [1,2].

While ruminant livestock undoubtedly generate sizable methane (CH_4_) emissions, their farming also plays a role in significantly affecting the release of gases impacting the climate. Ruminants contribute CH_4_ gas emissions from anaerobic enteric fermentation processes in the rumen [3]. Every year, domesticated ruminant animals collectively generate approximately 86 million metric tons (Tg) of CH_4_ [4,5]. According to Saunois et al. [6], the estimated CH_4_ emissions from ruminants were approximately 111 (106–116) Tg CH_4_/year, making up slightly over 30% of the total global emissions from 2008 to 2017. During the rumen’s feed degradation process, the production of acetate (C_2_) and butyrate (C_4_) production leads to pure hydrogen generation as a by-product. However, propionate (C_3_) formation offers an alternative pathway for hydrogen utilization, resulting in the production of CH_4_. In addition, ruminal methanogenic archaea also contribute to the formation of CH_4_, utilizing hydrogen and carbon dioxide (CO_2_) to generate energy [7,8].

Ruminant productivity can also be enhanced by reducing CH_4_ emissions and restricting greenhouse gas (GHG) emissions [9]. There have been studies on the strategy of employing multiple feed additives such as flavonoids, tannin, saponin, essential oils, enzymes, 3-nitrooxypropanol, organic acids, and lipids for purposes of reducing CH_4_ emissions from ruminant livestock [10–18]. Feeding ruminant tree leaves has been investigated, and multiple investigations have revealed a reduction in the creation of CH_4_. Furthermore, many experts have suggested tree leaves as a viable alternative animal protein source [19].

The marigold plant (*Tagetes erecta L.*) is acknowledged for its potential to treat wounds caused by bacterial infections. Due to the phytochemicals derived from marigolds possessing antibacterial qualities, their application has historically been utilized in treating injuries. These compounds effectively combat bacterial infections and promote wound healing [20]. It is a widely distributed plant worldwide and has been used as a traditional medicine in China [21]. It has been discovered that certain marigold species offer therapeutic qualities. They are capable of addressing numerous ailments, including such issues as irregular menstruation, varicose veins, hemorrhoids, burns, skin diseases, wounds, and duodenal ulcers [22]. Furthermore, the leaf part of the marigold has potential as a feed supplement because of its greater availability than the flowers [23]. Additionally, based on initial research, we identified that marigold leaves (MGLs) had 83.68% organic matter (OM), 16.87% crude protein (CP), 15.83% crude fiber (CF), 2.17% ether extract (EE), 48.81% nitrogen-free extract (NFE), and 64.25% total digestible nutrient (TDN) content. This diverse array of active natural antioxidants, vitamins, terpenoids, alkaloids, phenolics, flavonoids, saponins, and steroids are all together within MGL, working harmoniously. These components make MGL valuable as a feed additive for various purposes [24]. While ruminants are recognized as a significant provider of high-quality animal protein, they are also accountable for considerable GHG. However, natural feed additives available in the form of secondary plant metabolites can modify ruminal enzyme activities and fermentation, effectively reducing CH_4_ production and enhancing ruminant production. These additives offer a promising alternative to address the environmental effects of ruminant farming while improving overall productivity [25].

Several researchers have examined the advantages of using marigolds for animals. Hou et al. [26] investigated the quality improvement of marigold crop residues using lactic acid bacteria. Rahman et al. [27] concluded that MGL paste reduced tissue reactions and promoted effective wound healing in sheep. Wencelová et al. [28] studied medicinal plant mixtures containing marigold flowers as sheep feed and showed increased nutrient digestibility and decreased CH_4_. Nevertheless, there is currently a scarcity of data on the specific impacts of implementing MGL in ruminant feeding, particularly concerning ruminal fermentation.

MGL as a feed supplement shows the potential to mitigate CH_4_ production and enhance rumen fermentation in ruminants. Incorporating MGL into the diet can improve sustainability and environmental impact by mitigating CH_4_ emissions, a potent greenhouse gas. The beneficial effects of rumen fermentation lead to increased nutrient utilization and the potential for increased animal performance. Additional investigations are needed to explore the ideal dose and long-term impact of MGL use in ruminant rations. Therefore, this research aims to examine the use of MGL in diets and its effects on rumen enzyme activity, fermentation parameters, CH_4_ emissions, and nutrient digestibility *in vitro*. The results obtained from *in vitro* studies can serve as a basis for further investigations and inform the potential application of MGL in ruminant nutrition.

## Materials and Methods

### Ethical statement

The animal ethics committee at Universitas Gadjah Mada approved all procedures used in the current trial (No. 003/EC-FKH/Eks./2023).

### Experimental diets

The experimental setup was a complete randomized design with four treatments, each having six replications (*n* = 6). The experimental diets comprised different proportions of MGL incorporated into the dry matter (DM) rations. The treatments include 0% MGL inclusion (referred to as MGL-0), 7% MGL inclusion (referred to as MGL-7), and 14% MGL inclusion (referred to as MGL-14). Fresh MGL was collected 6 weeks after sowing. *Pennisetum purpureum* (elephant grass) and concentrate containing palm kernel meal, wheat bran, rice bran, soybean meal, molasses, and corn cob were used as basal feed. The composition of the diet for each experimental group is presented in Table 1. The diets were prepared to meet Bali cattle’s needs with 12% CP and 57% TDN [29].

In this study, samples of MGL and fresh elephant grass were dried at 55°C for 3–4 days until a constant weight was achieved (with a water content of approximately 10%–15%). Once dried, the samples were crushed into a reasonably acceptable particle size using a Willey mill with a screen size of 1 mm. Implementing this process guaranteed consistency and enabled precise chemical analysis of the samples. Furthermore, the ground samples were utilized in the *in vitro* test. The feed chemical constituents, consisting of DM, OM, CP, CF, EE, and NFE (as shown in Table 1), were determined using the procedures of AOAC [30]. The secondary metabolites in the MGL sample were determined using the UV-Vis spectroscopy method, as outlined by Mabasa et al. [31].

### Rumen liquor preparation and in vitro fermentation

In the experiment, two Bali cattle fitted with rumen fistulae were served as rumen liquor, a source of microbial populations and enzymes for *in vitro* rumen fermentation studies. Throughout the adaptation period, which lasted for 14 days, the animals were provided with a concentrate with a CP content of 13% and a TDN content of 75%. The livestock were fed twice daily (morning and afternoon), with the amount provided being 3% of their body weight. The grass was provided *ad libitum* to the cattle and given unlimited access to water. Fresh rumen fluid was taken from the animals in the morning before feeding. The collected liquor was then filtered using four layers of filter cloth to remove any solid substances. Subsequently, the filtered rumen liquor was transferred to a thermally insulated container and maintained at 39°C. The container was taken to the laboratory for additional analysis within 15 min.

Therefore, a combination of rumen liquid and buffer medium was created at a ratio of 1:2 [32]. All mixing procedures were performed in anaerobic conditions to maintain rumen microbes in good condition according to their natural habitat. The feed substrate was weighed at 300 mg of DM for each dietary treatment. The weighed substrate was then transferred into a 100-ml glass syringe (Fortuna, Poulten & Graft GmbH, Germany). The pistons on each syringe were lubricated with Vaseline. Each glass syringe was supplied with 30 ml of rumen liquor medium while ensuring CO_2_ flushing. A total of 24 glass syringes were used in the experiment, with 6 syringes assigned to each of the 3 feed types. Additionally, six syringes were designated as blanks, containing only rumen fluid. All syringes were subjected to incubation at 39°C for 48 h.

### Fermentation gases and end-products

The gas measurements, which had been carefully recorded at pre-determined checkpoints along a timeline stretching from the onset of incubation to 2 days beyond its conclusion—precisely at 0, 1, 2, 4, 6, 8, 10, 12, 24, and 48 h—were undertaken with scrupulous attention to detail to monitor the amounts being generated throughout the various phases. At 48 h post-incubation, the rumen fluid from each syringe was strained using a glass wool crucible to prepare the samples for further analysis. This filtration helped remove any solid particles or debris in the fluid. After that, the solution was spun for 15 min at 3,000 g in a centrifuge. This centrifugation step separated the liquid phase from any remaining sediment or particulate matter, facilitating subsequent analyses or measurements of the clarified rumen fluid. The obtained filtrate was subsequently utilized for pH analysis through a pH meter (Hanna Model H1-2210). The procedures outlined in the works of Azizi et al. [33] to quantify the functions of carboxymethyl cellulase (CMC-ase), amylase, and protease were tracked sequentially.

**Table 1.** Dietary ingredients and chemical composition of experimental diet.

Item	Treatment			Concentrate	Elephant grass	MGLs
	MGL-0	MGL-7	MGL-14			
Ingredients (%)						
Palm kernel meal	2.40	2.40	2.40	-	-	-
Wheat bran	6.30	6.30	6.30	-	-	-
Rice bran	10.50	10.50	10.50	-	-	-
Soybean meal	3.00	3.00	3.00	-	-	-
Molasses	1.05	1.05	1.05	-	-	-
Corncob	6.75	6.75	6.75	-	-	-
Elephant grass	70.00	63.00	56.00	-	-	-
MGLs	0.00	7.00	14.00	-	-	-
Chemical composition						
DM, %	92.11	91.79	91.48	89.04	93.42	88.93
% of DM						
OM	87.23	87.03	86.84	88.94	86.50	83.68
CP	11.40	11.89	12.39	15.28	9.73	16.87
CF	31.97	30.50	29.04	20.71	36.80	15.83
EE	1.76	1.83	1.89	2.85	1.30	2.17
Ash	12.77	12.97	13.16	11.06	13.15	16.32
NFE	42.10	42.81	43.52	50.10	38.67	48.81
TDN	56.99	57.75	58.50	65.24	53.46	64.25

MGL-0 = diets supplemented with 0% of the marigold leaves (control); MGL-7 = diets supplemented with 7% of the marigold leaves; MGL-14 = diets supplemented with 14% of the marigold leaves.

The procedure defined in the study performed by Wang et al. [34] was used to analyze volatile fatty acids (VFAs). The spectrophotometry method assessed the ammonia-nitrogen (NH_3_–N) and microbial protein contents, while total protozoa were enumerated using a Neubauer property [35]. After a 48-h incubation period, a 10 ml volume of gas was retrieved from the airspace inside the syringe. This extraction was carried out using a leakproof syringe to ensure accurate sampling. The extracted gas samples were then tested using a Shimadzu GC-14B gas chromatograph to test for levels of CH_4_ production [36].

### Nutrient digestibility

The nutrient digestibility was determined in two phases, each with different incubation periods. The initial stage necessitates incubating for 48 h, whereas the subsequent phase lengthens the incubation period to 96 h. In the first step, the 50 ml test tubes containing 250 mg DM feed substrate were prepared for rumen digestibility analysis. Then, it was added to McDougall’s artificial saliva solution and rumen liquid (4:1, proportion ratio). A 25 ml medium solution was utilized to analyze DM and OM digestibility, whereas a 50 ml solution was used to assess CP and CF digestibility. Some tubes were left empty without feeding substrate as blanks. Each tube was purged with CO_2_ for each treatment to maintain anaerobic conditions and then incubated at 39°C. Following a 48-h incubation period, the liquid and feed substrate were separated using a glass wool crucible. The feed substrate obtained was utilized to assess the nutrient digestibility of the rumen. During the second step of the process, individual vessels holding the material under examination were augmented with a combination of 20% hydrochloric acid and 5% pepsin enzyme at a ratio of three-part acid to one-part enzyme. Afterward, these tubes were further incubated for an additional 48-h. Following the second step, the feed substrate underwent filtration, and the remaining substrate was subjected to analysis to ascertain the total digestibility of DM, OM, CP, and CF [37,38].

### Statistical analysis

The current research employs an equation model to measure gas production’s kinetics. This model is commonly used for analyzing the patterns and rates of gas production in ruminal fermentation experiments [39].

*Y* = *a* + *b* (1 − *e*^−*ct*^)

The parameters used for analysis were defined as follows: *a*-fraction, the fraction that dissolves immediately (ml); *b*-fraction, the fraction that is insoluble but can be degraded (ml); *c*-fraction, the rate at which degradation occurs (ml/h); *a* + *b* fraction, the overall gas production potential (ml); *t*, the length of the incubation period (h); *y*, the quantity of gas generated at a given time “*t*” (ml). The collected data underwent an analysis of variance using SAS On Demand for Academics^®^ Software (www.sas.com) for statistical analysis. Following that, a comparison of means was performed using the Duncan multiple range test at a significance level of *p <* 0.05.

## Results

### Secondary metabolites in MGL

The secondary metabolites of MGL are presented in Table 2. The concentrations of various secondary metabolites were calculated in milligrams per gram (mg/gm) based on the percentage of DM. The flavonoid content exhibited the highest value (167.31 mgQE/gm), followed by total phenols (39.69 mgGAE/gm), total tannins (32.50 mgTAE/gm), and saponins (10.91 mg/gm), respectively. Moreover, total tannin consists of condensed tannins (20.58 mgTAE/gm) and hydrolyzed tannins (11.92 mgTAE/gm), while the non-tannin phenolics observed on MGL were 7.20 mgGAE/gm. Other constituents of MGL were steroid 7412.44 µg/gm, alkaloid 6963 µg/gm, and ascorbic acid 68.90 µg/gm.

**Table 2.** The secondary metabolites of MGLs.

Item	Value
Flavonoids (mgQE/gm)	167.31
Total phenolic (mgGAE/gm)	39.69
Total tannin (mgTAE/gm)	32.50
Condensed tannin (mgTAE/gm)	20.58
Hydrolysable tannin (mgTAE/gm)	11.92
Non-tannin phenolic (mgGAE/gm)	7.20
Saponin (mg/gm)	10.91
Steroid (µg/gm)	7,412.44
Alkaloid (µg/gm)	6,963.96
Ascorbic acid (µg/gm)	68.90

### Ruminal enzyme activities

As indicated in Table 3, the ruminal CMC-ase, amylase, and protease activity were not influenced by MGL-7 and MGL-14 treatment in comparison to MGL-0 (*p >* 0.05).

### Cumulative gas production, kinetics, and methane production

Dietary supplementation with MGL did not modify (*p >* 0.05) the cumulative gas production (Table 4). In addition, the kinetic parameters, such as the *a*, *b*, *a* + *b*, and* c* fractions, did not show any significant variations (*p* > 0.05) across the dietary groups (Table 4). Moreover, when evaluated in milliliters or milliliters per unit of digested DM or OM, there were no differences (*p* > 0.05) in ruminal CH_4_ and CO_2_ production across the dietary treatments (Table 4).

### Rumen fermentation parameters

The utilization of MGL in the rations significantly affected (*p <* 0.05) the ruminal fermentation parameters, as shown in Table 5. The microbial protein content significantly improved (*p* < 0.05) with 7% and 14% MGL added to the rations instead of the MGL-0 treatment; meanwhile, a significant increase in NH_3_–N levels (*p* < 0.05) was observed in the 14% MGL group. Additionally, the MGL-7 treatment decreased (*p <* 0.05) the total protozoa and MGL-14 groups relative to the MGL-0. Among the dietary treatments, the MGL-14 group exhibited the highest total VFA, as well as molar proportions of C_2_, C_3_, C_4_, and the C_2_:C_3_ ratio. However, the ruminal pH value was not influenced (*p >* 0.05) by the MGL levels.

### Nutrient digestibility

**Figure 1.**
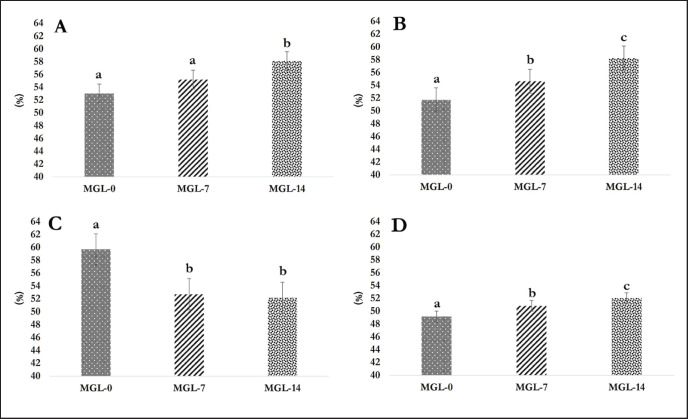
Ruminal digestibility as affected with the MGL inclusion in the diet (%). (A) DM digestibility; (B) OM digestibility; (C) CP digestibility; and (D) CF digestibility.

**Figure 2.**
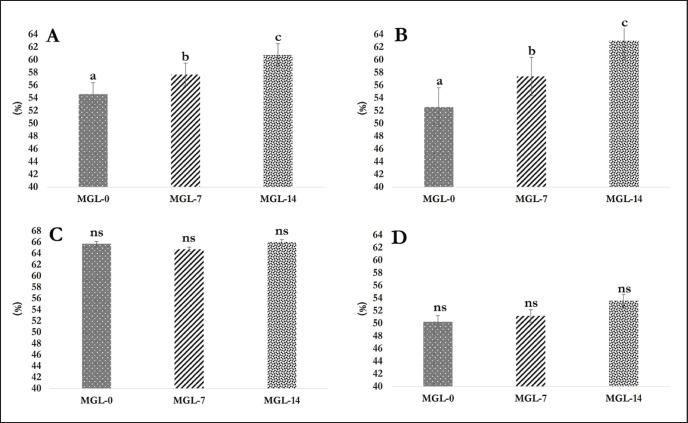
Total digestibility as affected with the MGL inclusion in the diet (%). (A) DM digestibility; (B) OM digestibility; (C) CP digestibility; and (D) CF digestibility.

DM, OM, and CF digestibility in the rumen revealed a significant improvement (*p <* 0.05) for the inclusion of MGL-14 and MGL-7 compared to MGL-0 (Fig. 1). Including both 7% and 14% MGL significantly declined (*p <* 0.05) the CP digestibility in the rumen compared to MGL-0. The total DM and OM digestibility were greater (*p <* 0.05) in the MGL-14 and MGL-7 groups than in the control group. Nevertheless, the utilization of MGL in the diets had no significant impact (*p >* 0.05) on total CF and CP digestibility, as depicted in Figure 2.

**Table 3.** Ruminal enzyme activities as affected by the MGLs inclusion in the diet.

Item	Treatment			SEM	*p*-value
	MGL-0	MGL-7	MGL-14		
CMC-ase (U/gm)	5.60	6.09	5.48	0.148	0.212
Amylase (U/gm)	16.58	15.77	14.18	0.809	0.514
Protease (U/gm)	27.47	28.79	29.23	0.592	0.495

CMC-ase = carboxymethyl cellulase; MGL-0 = diets supplemented with 0% of the marigold leaves (control); MGL-7 = diets supplemented with 7% of the marigold leaves; MGL-14 = diets supplemented with 14% of the marigold leaves; SEM = standard error of the mean.

## Discussion

### Secondary metabolites in MGL

Leaves from various tree species have been identified as valuable feed sources for ruminant animals throughout the year, offering higher levels of essential nutrients and bioactive compounds than grasses [40]. While bioactive substances from plants may contain antinutritional qualities, their properties could also modulate ruminal fermentation procedures in a regulatory manner. Consequently, extensive studies have used bioactive compounds as natural feed supplements to exploit rumen fermentation processes. Specifically, the impacts of flavonoids and tannin as dietary supplements in animal diets, particularly for ruminant species, have gained increasing attention and investigation [41].

Specific secondary metabolites within MGL, including flavonoids, phenolics, tannins, and saponins, may enhance the digestibility of nutrients due to their possible effects. These compounds can serve as energy sources for rumen microbes without adversely affecting the fermentation processes in the rumen. Moreover, they possess protozoan defecation effects, which aid in reducing CH_4_ production and increasing C_2_ production. These effects contribute to improved ruminant carbohydrate digestion [42]. Furthermore, secondary metabolites could potentially alter the cellulolytic and NH_3_–N-producing bacteria in the rumen. They can influence the composition and activity of these microbial populations, thereby affecting the breaking of cellulose and protein digestion. Furthermore, these metabolites could curb the gas production essential for the process of methanogenesis, thereby potentially resulting in lowered CH_4_ emissions from ruminant livestock [43].

**Table 4.** Rumen fermentation parameters as affected by the MGLs inclusion in the diet.

Item	Treatment			SEM	*p*-value
	MGL-0	MGL-7	MGL-14		
pH	6.50	6.53	6.56	0.021	0.567
NH_3_–N (mg/100 ml)	37.85^a^	42.31^ab^	46.30^b^	1.582	0.038
Microbial protein (mg/ml)	22.03^a^	31.71^b^	37.20^c^	1.595	0.001
Protozoa (10^5^ cells/ml)	1.38^a^	1.18^b^	0.90^c^	0.062	0.001
Total VFA (mM)	35.83^a^	45.56^b^	63.27^c^	3.003	0.001
C_2_ (mM)	18.26^a^	21.17^a^	34.88^b^	1.913	0.001
C_3_ (mM)	12.43^a^	15.84^b^	21.54^c^	0.274	0.001
C_4_ (mM)	5.36^a^	6.58^b^	7.68^c^	1.032	0.001
C_2_:C_3_	1.47^a^	1.54^a^	1.74^b^	0.678	0.001

^a–c^Means with different superscript within the same column are statistically significant differences (*p* < 0.05).

NH_3_–N = ammonia-nitrogen; VFA = volatile fatty acids; C_2_ = acetate; C_3_ = propionate; C_4_ = butyrate; MGL-0 = diets supplemented with 0% of the marigold leaves (control); MGL-7 = diets supplemented with 7% of the marigold leaves; MGL-14 = diets supplemented with 14% of the marigold leaves; SEM = standard error of the mean.

**Table 5.** Cumulative gas, kinetics, and methane (CH_4_) production as affected by the MGLs inclusion in the diet.

Item	Treatment			SEM	*p*-value
	MGL-0	MGL-7	MGL-14		
Cumulative gas (48 h) ml/300 mg DM	61.17	62.68	63.38	0.51	0.198
Gas kinetics					
a-fraction (ml/300 mg DM)	−5.60	−5.44	−5.71	0.19	0.862
b-fraction (ml/300 mg DM)	75.57	74.99	72.35	0.72	0.160
a + b-fraction (ml/300 mg DM)	79.76	78.10	77.36	0.84	0.566
c-fraction (ml/h)	0.048	0.051	0.052	0.00	0.398
CH_4_ Production					
CH_4_ (ml/300 mg DM)	6.187	6.27	6.10	0.084	0.699
CH_4_ [ml/digested DM (mg)]	0.04	0.04	0.05	0.002	0.651
CH_4_ [ml/digested OM (mg)]	0.04	0.05	0.05	0.002	0.060
CO_2_ (ml/300 mg DM)	39.77	41.82	42.56	0.774	0.345
CO_2_ [ml/digested DM (mg)]	0.26	0.26	0.29	0.006	0.346
CO_2_ [ml/digested OM (mg)]	0.26^a^	0.34^b^	0.35^b^	0.010	0.001

^a–b^Means with different superscript within the same column are statistically significant differences (*p* < 0.05).

a-fraction = the fraction that dissolves immediately; b-fraction = the fraction that is insoluble but can be degraded; c-fraction = the rate at which degradation occurs e; a + b-fraction = the overall gas production potential; MGL-0 = diets supplemented with 0% of the marigold leaves (control); MGL-7 = diets supplemented with 7% of the marigold leaves; MGL-14 = diets supplemented with 14% of the marigold leaves; SEM = standard error of the mean.

### Ruminal enzyme activities

The inclusion of MGL at various levels did not influence the ruminal enzyme activities of CMC-ase, protease, and amylase. Because the lower concentrations of tannins and saponins within MGL lacked detrimental impacts on ruminal enzyme functions, these results possess a potential rationale. According to Jadhav et al. [44], low levels of secondary metabolites in feed had no harmful consequences for rumen enzyme activity. While their antimicrobial capabilities could, at relatively elevated levels, induce a diminution of enzymatic function, Abdel-Raheem and Hassan [45] likewise determined that supplementing the diet of buffalo calves with 15% or 20% *Moringa oleifera* leaves, which themselves contain comparable phytochemicals such as flavonoids, phenolics, tannins, and saponin, served to decrease the enzymatic activities within the rumen of cellulase, protease, and amylase. In addition, the variations in substrates and bioactive components in different plant species can contribute to differences in research results.

### Rumen fermentation parameters

No effect of MGL supplementation on the ruminal pH was observed. The pH values observed for the treatment groups in the rumen, ranging from 6.50 to 6.56, were consistent with the normal range of 6.3–7, typically reported in similar studies by Reis et al. [46]. The NH_3_–N was higher when supplemented with MGL at 14% DM when compared with the control. Likewise, the research undertaken by Jafari et al. [47] had comparable findings; it was noticed that NH_3_–N levels were highest in the 31.25 mg/250 mg DM supplementation group when added with papaya leaf containing phenolics (30.31 GAE/gm). The higher concentrations of NH_3_–N demonstrated in the MGL groups could be attributable to the activity of hyper-ammonia-producing bacteria, which contribute to higher amino acid deamination. It is implied from this that the existence of MGL could potentially incite microbes that specialize in generating more ammonia as an unintended result of amino acid processing [48]. The NH_3_–N is utilized for microbial protein synthesis. Previous studies, such as McDonald et al. [49], have illustrated that the concentration of NH_3_–N in ruminal fluid typically ranges from 8.5 to 30 mg/100 ml. However, it is worth noting that excess NH_3_–N exceeding 50 mg NH_3_–N/l does not significantly impact microbial protein synthesis and is ultimately excreted, as indicated by Neto et al. [50].

The increase in MGL dose is linear with increasing microbial protein concentrations. In contrast with the control group, the microbial proteins were augmented to a substantially greater extent within the MGL-7 and MGL-14 groups, with respective increases of 43.87% and 68.83% from the baseline measure. This can be expected because using MGL-containing flavonoids could affect the rumen microbial population, which can affect the microbial protein produced. At the same time, flavonoids are understood to have the ability to govern the makeup of microbes residing in the rumen, potentially bringing about shifts in nutrient breakdown metabolism [51]. This could also be linked to a reduction in the protozoan population when MGL was included in the ration. Morsy et al. [52] hypothesize that the decrease in total rumen protozoa counts seen in diets containing secondary plant compounds may help to explain the higher rumen bacterial counts. When protozoan populations decline, the rumen environment becomes more conducive to the growth and proliferation of bacteria. Protozoa are known to prey on bacteria; reducing their numbers increases nutrition availability while lessening predation pressure on bacteria. As a result, the rumen’s microbial population might grow, increasing the amount of rumen bacteria. According to Patra and Saxena [53], secondary metabolites, particularly saponins, substantially affect the rumen by inhibiting protozoa. This inhibition could promote the synthesis process of microbial proteins and facilitate the transportation of proteins to the duodenum. It is essential to consider that protein fermentation in the rumen generates NH_3_–N as the end product, which plays a vital role in synthesizing microbial protein within the rumen.

The study examined how the chemical makeup of flavonoids might be connected to their biological effects on different types of cells, shedding light on a potential correlation between flavonoid composition and cellular impact [54]. Researchers discovered through their investigation the antimalarial abilities of flavonoids and precisely determined distinct biochemical objectives inside the Plasmodium falciparum parasite. Additionally, the catechins were found to interact with specific molecular targets, such as enzymes connected to the biosynthesis of fatty acids. Another study by Kim et al. [55] reported that flavonoids reduced ciliate protozoan populations by over 60%. Ciliate protozoa have a significant role in methanogenesis, where methanogenesis can live symbiotically attached to the protozoa’s surface. Therefore, flavonoids in the diets decreased ciliate-associated methanogens, ultimately reducing CH_4_ emissions.

The supplementation of MGL at 14% DM increased the total VFA, C_2_, C_3_, and C_4_. These findings contrast with those of Bryszak et al. [56], who showed that giving dairy cows 2 kg/day of flavonoids- and tannin-rich black currant seed residues did not change their VFA profiles. While the effect of flavonoid supplementation on VFA formation may vary depending on the flavonoid constituent utilized, the findings suggested this impact relied on the specific component employed in the supplementation. Berchez et al. [57] reported contrasting findings, suggesting increased specific VFA concentrations. This phenomenon can be explained by the capability of rumen microbes to adjust to phenolic properties and flavonoids over time, which may result in the conversion of highly toxic phenolic acids into less harmful forms through hydrogenation. While the adaptation of microbes may add to the antimicrobial qualities of flavonoid mixtures due to their chemical makeup, the precise means rely on their inherent constitution [58]. Including MGL in rations has increased total VFA, C_2_, and C_3_, indicating improved ruminal fermentation and feed digestion. This observation is corroborated by the results of Kholif and Olafadehan [59], indicating that secondary metabolites in plants can enhance the proportion of C_3_ and, in some cases, C_2_ due to an enhanced breakdown of both complex and simple sugars during the digestive process. This suggests that the secondary metabolites in MGL can positively influence rumen fermentation and promote more efficient utilization of carbohydrates in ruminants.

The reduction in rumen protozoa counts seen with MGL inclusion in the diet could be attributed to secondary metabolites in MGL. Other secondary metabolites found in plants, such as saponins, have been proven to eliminate rumen protozoa. Tannins, in particular, have long been recognized for their potent defaunation effects or the ability to diminish or eliminate rumen protozoan populations. Although the precise mechanism by which tannins create these effects is unknown, further research is required [60].

### Cumulative gas production, kinetics, and methane emission

The incorporation of MGL in the rations did not influence the cumulative gas production or kinetic profiles. The lack of a substantial change in cumulative gas production when MGL-containing flavonoid levels increased suggests that MGL has a minimal impact on the rumen fermentation rate. This implies that including MGL in the diet does not adversely affect ruminal fermentation. Oskoueian et al. [61] exhibited that some flavonoid types (naringin, rutin, and quercetin) increased gas production, while flavones and myricetin decreased *in vitro* cumulative gas. While the influence of flavonoids on gas production fluctuated, these divergences seemed explicable by considering the particular substrate selected for use during the fermentation procedure. Different substrates may interact differently with flavonoids, resulting in diverse effects on gas production and fermentation parameters. Thus, substrate selection plays a vital role in defining the specific impact of flavonoids on rumen metabolism. Flavonoids’ effect on fermentation is also influenced by dosage, type of phytofactors, molecular weights, and the specific substrate employed. Based on the present findings, using MGL-containing flavonoids rather than pure flavonoids alone has a noticeable impact on cumulative gas production and kinetics.

In this research, the utilization of MGL in diets led to a decline in the protozoan population. Unfortunately, the outcome did not lead to a diminishing of the CH_4_ quantity as one might have expected. The results Goel et al. [62] found were consistent with what was obtained. Incorporating *Sesbania sesban* leaves and Fenugreek seeds, which contain secondary metabolites, into the sheep’s diet reduced the total protozoa count within the rumen. However, this supplementation demonstrated no discernible effect on CH_4_ production. According to Patra et al. [63], the correlation between protozoa and methanogenesis is intricate and can be affected by some variables. One factor in this difference is the contrasting generation times between single-celled protozoa and their methanogen counterparts. Changes in methanogenic population composition may play a role in these dynamic interactions. Although the interaction between protozoa and methanogen is complicated and warrants further investigation, these considerations indicate that their interactions could be more complex. Although Goel and Makkar [64] explained that protozoa reduction trials did not always result in lower CH_4_ emissions, more research is required to properly comprehend the complex interactions between different microbial species. This can be caused by species-independent methanogenic bacteria that can survive or modify the efficiency of CH_4_ synthesis. A decline in the protozoa population could change the microbial community structure within the rumen. Specifically, any reduction in the *Methanobacteriaceae* species commonly associated with protozoa could be offset by a rise in free-living *Methanobacteriales*. Consequently, this shift in the composition of the microbial community can facilitate higher levels of interspecies hydrogen transfer. The increased population of free-living *Methanobacteriales* may interact more intensively with hydrogen-producing bacteria, such as *Ruminococcus flavefaciens* and *Fibrobacter succinogenes*. However, despite these changes, the overall effect on CH_4_ production may be negligible or insignificant.

### Nutrient digestibility

The inclusion of MGL in diets significantly impacted nutrient digestibility. Feeding MGL increased the rumen’s digestibility of DM, OM, and CF. However, the ruminal CP digestibility declined when MGL was included in the rations. This impact can be attributed to the rumen microflora’s capacity to metabolize the secondary metabolites present in MGL, including flavonoids, phenolics, tannins, and saponins, as sources of energy without causing any detrimental effects on the ruminal fermentation process [65].

As mentioned earlier, secondary metabolite properties in MGL can stimulate the growth and activities of ruminal fibrolytic microbes. This stimulation leads to a faster degradation rate and a greater extent of substrates in the rumen [66]. *Moringa oleifera* leaves, which contain similar phytochemicals (i.e., flavonoids, phenolics, tannin, and saponin), improved the DM, OM, and fiber digestibility [67]. *Emblica officinalis* fruit pomace, with its phenolic compounds (224 gm/kg DM), exhibited increased OM and neutral detergent fiber digestibility with a 10 gm/kg dosage. The analysis of MGL for tannins and saponins demonstrated concentrations of 32.50 mgTAE/gm and 10.91 mg/gm, respectively. These concentrations were below the critical levels that could hinder ruminal fermentation and feed digestibility. This suggests that the levels of tannins and saponins in MGL are within a safe range and unlikely to have detrimental effects on ruminal processes and feed nutrient content utilization. The decline in CP digestibility in phenolic compounds and tannins found in MGL may be ascribed to their ability to bind with proteins, thereby reducing their degradation by ruminal microbes. Tannins and phenolic compounds have been known to interact with proteins, resulting in complex forms, making them less accessible to microbial enzymes and hindering their breakdown. This can result in a decrease in CP digestibility in the rumen [68].

The weakness in this research, which suggests further research, is the need for specific detection of rumen microbes (microbiome), especially microorganisms involved in forming CH_4_ gas in the rumen. In addition, it is necessary to conduct further research using *in vivo* methods in livestock to validate the optimal level of MGL usage.

## Conclusion

The present study suggests that using MGL until 14% DM in diets can enhance ruminal fermentation parameters and nutrient digestibility *in vitro* without negatively affecting gas production kinetics or ruminal enzyme activities. Furthermore, CH_4_ production was not influenced by MGL supplementation. Nevertheless, additional research is required to explore the utilization of MGL in live animal trials.

## List of abbreviations

µg, microgram; a + b fraction, the overall gas production potential; a-fraction, the fraction that dissolves immediately; b-fraction, the fraction that is insoluble but can be degraded; c-fraction, the rate at which degradation occurs; C, celsius; C_2_, acetate; C_3_, propionate; C_4_, butyrate; CF, crude fiber; CH_4_, methane; CMC-ase, carboxymethyl cellulase; CO_2_, carbon dioxide; CP, crude protein; DM, dry matter; EE, ether extract; gm, gram; g, gravitation; GAE, gallic acid equivalent; GHG, a greenhouse gas; h, hour; l, liter; mg, milligram; MGL, marigold leaf; ml, milliliter; mm, millimeter; NFE, nitrogen-free extract; NH_3_–N, ammonia-nitrogen; OM, organic matter; QE, quercetin equivalent; t, the length of the incubation period; TAE, tannic acid equivalent; TDN, total digestible nutrient; Tg, metric ton; U/gm, unit per gram; VFA, volatile fatty acids; y, the quantity of gas generated at a given time.
